# Fulminant streptococcal toxic shock syndrome presenting as cervical epidural abscess: a case report

**DOI:** 10.12701/jyms.2026.43.38

**Published:** 2026-06-09

**Authors:** Su Jeong Shin

**Affiliations:** Department of Emergency Medicine, Yeungnam University College of Medicine, Daegu, Korea

**Keywords:** Disseminated intravascular coagulation, Epidural abscess, Group A *Streptococcus*, Streptococcal toxic shock syndrome, *Streptococcus pyogenes*

## Abstract

Streptococcal toxic shock syndrome (STSS), caused by Group A *Streptococcus*, is a life-threatening condition that can lead to organ failure within several days of symptom onset. Fulminant presentations resulting in death within hours of emergency department arrival are exceedingly rare and have a nearly universally fatal prognosis. Prompt recognition is critical; however, early clinical features may closely mimic those of other surgical emergencies, creating diagnostic challenges. A 75-year-old male was transferred who had posterior neck and bilateral shoulder pain for several days and a suspected cervical epidural abscess. The patient complained of dyspnea at the time of presentation. Despite early endotracheal intubation before the development of overt respiratory failure, the patient rapidly progressed to cardiac arrest and died. Blood cultures obtained at presentation were positive for *Streptococcus pyogenes* after 3 days, as reported in this case. Although spinal epidural abscesses are not a typical presentation of STSS, they can lead to rapid clinical deterioration in the early phase. Accordingly, a high index of suspicion and early diagnostic evaluation are crucial for timely management.

## Introduction

Invasive *Streptococcus pyogenes* (Group A *Streptococcus* [GAS]) infection has long been recognized as a potentially fatal condition that can progress to severe disease even in the absence of identifiable predisposing factors [1,2]. Since the beginning of the pandemic, GAS infections have emerged as a public health concern that extends beyond specific geographic regions [3-5]. The incidence appears to be increasing in certain age groups, including children and older individuals, and the disease increasingly presents diverse forms of severe infection beyond the classical manifestations of pharyngitis and skin or soft tissue infection, thereby posing a significant threat to patients [6-8].

A spinal epidural abscess is a distinct but relatively severe spinal emergency. *Staphylococcus* species are the most frequently identified causative organisms of spinal epidural abscesses. Treatment generally consists of surgical management, including procedures such as corpectomy or laminectomy, combined with appropriate antibiotic therapy [9,10]. Predisposing factors for spinal epidural abscesses include immunocompromised conditions such as acquired immunodeficiency syndrome, alcoholism, liver cirrhosis, and diabetes mellitus, as well as structural or procedural factors including spinal injury and prior spinal surgery [11,12].

Here, I report the case of a 75-year-old male without evidence of spinal injury, prior spinal surgery, or significant underlying comorbidities who was transferred to our emergency department with a working diagnosis of cervical epidural abscess based on magnetic resonance imaging (MRI) findings but was ultimately diagnosed posthumously with fulminant streptococcal toxic shock syndrome (STSS) caused by GAS. I present this case to highlight the potentially fatal consequences of this diagnostic pitfall and emphasize the need for early recognition of STSS, even when a structural etiology appears radiologically plausible.

## Case

**Ethics statement:** Written informed consent for publication was obtained from the patient’s legal representative.

A 75-year-old male presented to our emergency department around midnight with acute severe bilateral shoulder and posterior neck pain. He had a history of asthma and a minimally displaced left humeral fracture treated conservatively at an orthopedic clinic approximately 5 months earlier. His wife reported that he was generally independent and had good functional health before the event. He had experienced generalized malaise without definite upper respiratory symptoms (such as sore throat or cough) for several days preceding his arrival in the emergency department and had no prior history of neck injury.

On the morning of presentation, the severity of his neck and shoulder pain prompted him to revisit the orthopedic clinic where the humeral fracture had been managed. The attending orthopedic surgeon was concerned that the pain pattern was inconsistent with a glenohumeral or humeral etiology and ordered urgent cervical MRI. The MRI raised concerns about cervical epidural fluid collection (Fig. 1), and the patient was transferred to our tertiary emergency department for further evaluation and management. The patient’s clinical course from symptom onset to death is shown in Fig. 2.

Upon arrival at the emergency department, the patient was afebrile (36.0°C) with marked tachycardia (160 beats/minute, wide-complex), tachypnea (28 breaths/minute), oxygen saturation of 88% on nasal cannula at 3 L/minute, and an initial blood pressure of 200/100 mmHg. Arterial blood gas analysis immediately revealed metabolic acidosis with insufficient respiratory compensation. A 12-lead electrocardiogram confirmed wide-complex tachycardia. Given the severity of the hemodynamic compromise and clinical trajectory, the team elected for emergent airway management. After explaining the procedure to the patient’s family, a modified rapid sequence intubation was performed.

Immediately following endotracheal intubation, the patient deteriorated into cardiac arrest from pulseless electrical activity. Advanced cardiac life support was initiated within 20 minutes of emergency department arrival. Despite approximately 1 hour of sustained cardiopulmonary resuscitation with vasopressors, advanced resuscitation, and supportive measures, spontaneous circulation was not restored. Cardiac rhythm degenerated to asystole, and death was declared.

The blood samples drawn on arrival were processed after the patient’s death. These results were consistent with advanced sepsis, disseminated intravascular coagulation (DIC), and multiple organ dysfunction (Table 1). The combination of severe thrombocytopenia, markedly prolonged coagulation times (prothrombin time [PT]/international normalized ratio [INR] and activated partial thromboplastin time [aPTT]), elevated lactate dehydrogenase (LDH) activity, hepatic transaminase elevation, and rising creatinine levels was consistent with fulminant DIC superimposed on septic multi-organ failure. The levels of inflammatory markers (C-reactive protein, 25.33 mg/dL; procalcitonin, 20.2 ng/mL) were markedly increased, reflecting a massive systemic inflammatory response.

Regarding microbiological results, the analysis of two sets of blood cultures obtained at the time of arrival at the emergency department was completed 3 days postmortem. Both sets were positive for *S. pyogenes* (GAS). Antibiotic susceptibility testing demonstrated susceptibility to all the agents tested, consistent with the standard GAS phenotype. No *emm* typing was performed to define the M protein type.

## Discussion

To the best of our knowledge, this case documents one of the most fulminant presentations of STSS encountered in the literature, characterized by the near-simultaneous occurrence of septic shock, DIC, cardiac arrest, and death within approximately 20 minutes of emergency department arrival, with the total prehospital disease course spanning only hours from symptom onset. The deceptive initial diagnosis of a suspected cervical epidural abscess illustrates a critical pitfall: the misattribution of septic musculoskeletal pain to a structural surgical emergency.

STSS is defined by the Working Group on Severe Streptococcal Infections as GAS bacteremia with hypotension and two or more of the following: renal impairment, coagulopathy, hepatic dysfunction, acute respiratory distress syndrome, soft tissue necrosis, and erythematous rash [13]. Our patient met multiple criteria retrospectively: confirmed GAS bacteremia, severe coagulopathy (PT/INR, 1.76; aPTT, 80.3 seconds), hepatic involvement (aspartate aminotransferase, 140 IU/L; LDH, 1,288 IU/L), and profound thrombocytopenia (platelets, 41,000/µL). Although the creatinine level of 1.63 mg/dL did not strictly meet the Working Group threshold for renal impairment (≥2.0 mg/dL or twice the upper limit of normal), the STSS criteria were nonetheless fulfilled based on the remaining organ dysfunction criteria above. These findings were obtained only postmortem, suggesting that laboratory confirmation may trail the clinical course of ultra-fulminant STSS.

The presence of cervical epidural signal abnormalities on MRI requires careful interpretation. Postmortem confirmation of GAS bacteremia raises the possibility that the epidural MRI findings represent septic seeding of the epidural space as part of hematogenous dissemination, rather than a primary epidural abscess. A case was reported of a patient with multiple cervical spinal epidural abscesses, whose symptoms began with severe posterior neck pain and progressed to quadriplegia [14]. The lesion was not limited to the spine, as a retropharyngeal abscess and extensive paraspinal necrotizing fasciitis also accompanied it. Another case of myelitis with profound bilateral lower extremity weakness and urinary incontinence was reported, and GAS was isolated from the cerebrospinal fluid and blood [15]. As previously described, GAS is not a common pathogen causing epidural abscess [9,10,16]. Hematogenous spread following persistent or temporary bacteremia has been implicated as a mechanism. In typical spinal epidural abscesses, neurological deterioration is required in addition to severe back/neck pain and fever. However, in the present case, neurological symptoms were not prominent. Furthermore, given the suspected location of the abscess in the cervical spine, bilateral weakness or sensory deficits in the upper extremities were expected, and the absence of such findings represents a key distinguishing feature from classic spinal epidural abscesses.

A critical barrier to timely diagnosis of STSS in acute settings is the inherent delay in microbiological confirmation. Blood culture results typically require 48 to 72 hours, a timeframe that rendered the pathogen-specific diagnosis practically inaccessible in the present case, where GAS was confirmed only 3 days after death. In this context, clinical manifestations and laboratory parameters must serve as the primary basis for empirical antibiotic selection. Markedly elevated procalcitonin, a biomarker that rises within 2 to 4 hours of systemic bacterial infection, along with severe thrombocytopenia and coagulopathy, should strongly prompt empirical treatment for invasive bacterial sepsis regardless of whether a structural focus has been identified [17-19].

In this case, piperacillin-tazobactam was administered as the initial empirical antibiotic therapy, which was a reasonable choice given the broad coverage required for a suspected spinal epidural abscess. However, if STSS had been considered earlier, the addition of clindamycin would have been considered. Although beta-lactam antibiotics demonstrate excellent bactericidal activity against *S. pyogenes*, they lack the ability to suppress the ongoing toxin production mediated by streptococcal superantigens. In contrast, clindamycin has been shown to attenuate toxin production through the inhibition of protein synthesis and may improve outcomes in patients with STSS. Accordingly, this case highlights a limitation in the initial management and underscores the importance of early consideration of STSS in patients presenting with septic shock, with prompt initiation of empirical combination therapy comprising a penicillin-based antibiotic and clindamycin [20].

The patient’s epidemiological profile is noteworthy. Population-based surveillance data demonstrate that adults aged 65 years and older carry the highest incidence of invasive GAS infection and account for nearly one third of all cases and half of all related deaths, with increasing age identified as an independent predictor of mortality [21-23]. Advanced age is an independent predictor of mortality in invasive GAS, likely reflecting an age-associated decline in innate and adaptive immune responses, reduced physiological reserve, and diminished capacity to mount a compensatory response to a superantigen-mediated cytokine storm [21]. Notably, this vulnerability persists even in the absence of classical comorbidities such as diabetes mellitus, malignancy, and immunosuppression, as illustrated in the present case, where the patient had no significant medical history beyond asthma. Furthermore, although STSS can be triggered by subtle skin lesions, no apparent portal of entry was identified in the neck or shoulder region in this case. This highlights the importance of performing a thorough whole-body skin examination in patients presenting with unexplained severe pain or acute shock regardless of the presence of fever.

We have reported a case of fulminant STSS caused by *S. pyogenes* that mimicked a cervical epidural abscess. In the context of advanced DIC and multi-organ failure, death occurred within 20 minutes of arrival at the emergency department from refractory cardiac arrest due to pulseless electrical activity. Hemodynamic instability, severe thrombocytopenia, coagulopathy, and markedly elevated inflammatory markers at presentation are critical factors that mandate immediate systemic resuscitation and administration of empirical broad-spectrum antibiotics, regardless of whether a structural source has been identified. Greater awareness of this presentation pattern may enable earlier recognition and intervention in future cases.

## Figures and Tables

**Fig. 1.**
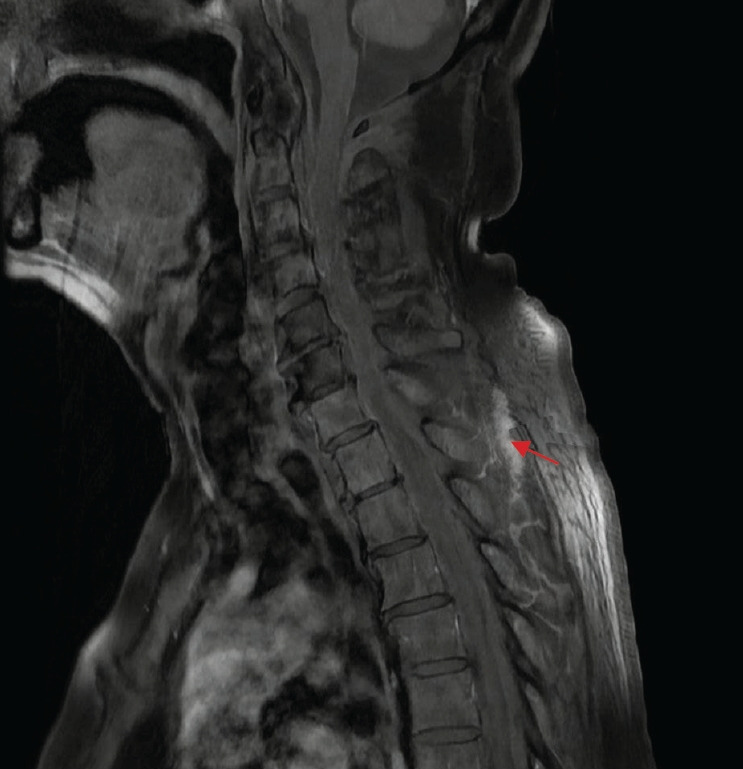
Cervical spine magnetic resonance imaging obtained at the referring hospital. T1-weighted post-contrast (gadolinium-enhanced) sagittal image demonstrating a hyperintense collection (arrow).

**Fig. 2.**
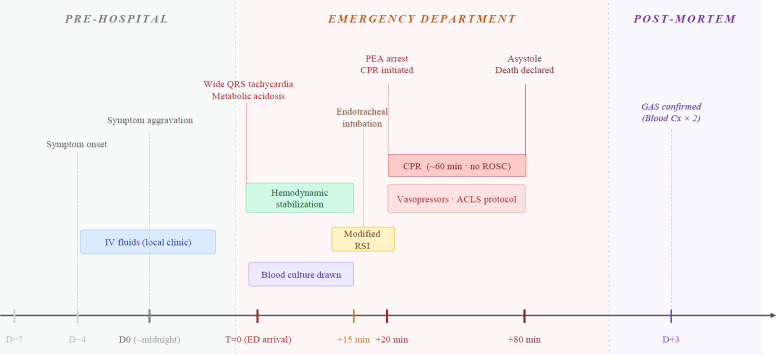
Clinical timeline of a fatal case of fulminant streptococcal toxic shock syndrome. PEA, pulseless electrical activity; CPR, cardiopulmonary resuscitation; GAS, Group A *Streptococcus*; IV, intravenous; RSI, rapid sequence intubation; ACLS, advanced cardiac life support; ROSC, return of spontaneous circulation; Cx, culture; ED, emergency department.

**Table 1.** Emergency department laboratory results

Parameter	Result	Reference range	Flag
WBC (K/µL)	9.04 (neutrophil, 86.4%)	4.0–10.0	–
RBC (M/µL)	4.04	4.5–5.5	Low
Hemoglobin (g/dL)	13.2	13.5–17.5	Low
Platelets (K/µL)	41	150–400	Low
CRP (mg/dL)	25.33	<0.5	High
Procalcitonin (ng/mL)	20.2	<0.5	High
AST (IU/L)	140	10–40	High
ALT (IU/L)	39	7–56	–
LDH (IU/L)	1,288	120–246	High
GGT (IU/L)	38	11–50	–
CPK (U/L)	165	30–200	–
BUN (mg/dL)	26.1	7–25	High
Creatinine (mg/dL)	1.63	0.7–1.3	High
PT (INR) (sec)	19.9 (1.76)	11.0–13.5	High
aPTT (sec)	80.3	25–35	High

WBC, white blood cell; RBC, red blood cell; CRP, C-reactive protein; AST, aspartate aminotransferase; ALT, alanine aminotransferase; LDH, lactate dehydrogenase; GGT, gamma-glutamyl transferase; CPK, creatine kinase; BUN, blood urea nitrogen; PT, prothrombin time; INR, international normalized ratio; aPTT, activated partial thromboplastin time.

## References

[1] Lamagni TL, Darenberg J, Luca-Harari B, Siljander T, Efstratiou A, Henriques-Normark B (2008). Epidemiology of severe *Streptococcus pyogenes* disease in Europe. J Clin Microbiol.

[2] Bessen DE (2009). Population biology of the human restricted pathogen, Streptococcus pyogenes. Infect Genet Evol.

[3] Goldberg-Bockhorn E, Hagemann B, Furitsch M, Hoffmann TK (2024). Invasive group A streptococcal infections in Europe after the COVID-19 pandemic. Dtsch Arztebl Int.

[4] Fukushima S, Saito T, Iwamoto Y, Takehara Y, Yamada H, Fujita K (2025). Trends in the growing impact of group A Streptococcus infection on public health after COVID-19 pandemic: a multicentral observational study in Okayama, Japan. Eur J Clin Microbiol Infect Dis.

[5] Leśnik P, Janc J, Biała M, Bartoszewicz M, Łysenko L, Słabisz N (2025). Old bug-new challenges after COVID-19 pandemic: severe invasive *Streptococcus pyogenes* infections in adults: a single-center experience in Poland. Pathogens.

[6] Russo DO, Moreira MM, Bentes AA, Marques BA, Carellos EV, Nogueira FD (2024). Invasive infections by group A Streptococcus infections in Brazil: a pediatric case series. Rev Paul Pediatr.

[7] Okuzono S, Ishimura M, Kanno S, Sonoda M, Kaku N, Motomura Y (2018). Streptococcus pyogenes-purpura fulminans as an invasive form of group A streptococcal infection. Ann Clin Microbiol Antimicrob.

[8] Malota M, Felbinger TW, Ruppert R, Nüssler NC (2015). Group A Streptococci: a rare and often misdiagnosed cause of spontaneous bacterial peritonitis in adults. Int J Surg Case Rep.

[9] Papadakis SA, Ampadiotaki MM, Pallis D, Tsivelekas K, Nikolakakos P, Agapitou L (2023). Cervical spinal epidural abscess: diagnosis, treatment, and outcomes: a case series and a literature review. J Clin Med.

[10] Turner A, Zhao L, Gauthier P, Chen S, Roffey DM, Wai EK (2019). Management of cervical spine epidural abscess: a systematic review. Ther Adv Infect Dis.

[11] Chao D, Nanda A (2002). Spinal epidural abscess: a diagnostic challenge. Am Fam Physician.

[12] Ju MW, Choi SW, Kwon HJ, Kim SH, Koh HS, Youm JY (2015). Treatment of spinal epidural abscess and predisposing factors of motor weakness: experience with 48 patients. Korean J Spine.

[13] (1993). Defining the group A streptococcal toxic shock syndrome. Rationale and consensus definition. The Working Group on Severe Streptococcal Infections. JAMA.

[14] Richardson C, Wattenbarger S (2021). A case report of quadriplegia and acute stroke from tracking retropharyngeal and epidural abscess complicated by necrotizing fasciitis. J Am Coll Emerg Physicians Open.

[15] Murphy KJ, Brunberg JA, Quint DJ, Kazanjian PH (1998). Spinal cord infection: myelitis and abscess formation. AJNR Am J Neuroradiol.

[16] Reihsaus E, Waldbaur H, Seeling W (2000). Spinal epidural abscess: a meta-analysis of 915 patients. Neurosurg Rev.

[17] Póvoa P, Coelho L, Dal-Pizzol F, Ferrer R, Huttner A, Conway Morris A (2023). How to use biomarkers of infection or sepsis at the bedside: guide to clinicians. Intensive Care Med.

[18] Velissaris D, Zareifopoulos N, Lagadinou M, Platanaki C, Tsiotsios K, Stavridis EL (2021). Procalcitonin and sepsis in the Emergency Department: an update. Eur Rev Med Pharmacol Sci.

[19] Vanderschueren S, De Weerdt A, Malbrain M, Vankersschaever D, Frans E, Wilmer A (2000). Thrombocytopenia and prognosis in intensive care. Crit Care Med.

[20] Stevens DL, Bisno AL, Chambers HF, Dellinger EP, Goldstein EJ, Gorbach SL (2014). Practice guidelines for the diagnosis and management of skin and soft tissue infections: 2014 update by the Infectious Diseases Society of America. Clin Infect Dis.

[21] Steer AC, Lamagni T, Curtis N, Carapetis JR (2012). Invasive group a streptococcal disease: epidemiology, pathogenesis and management. Drugs.

[22] Nelson GE, Pondo T, Toews KA, Farley MM, Lindegren ML, Lynfield R (2016). Epidemiology of invasive group A streptococcal infections in the United States, 2005-2012. Clin Infect Dis.

[23] Thigpen MC, Richards CL, Lynfield R, Barrett NL, Harrison LH, Arnold KE (2007). Invasive group A streptococcal infection in older adults in long-term care facilities and the community, United States, 1998-2003. Emerg Infect Dis.

